# Eftozanermin alfa (ABBV-621) monotherapy in patients with previously treated solid tumors: findings of a phase 1, first-in-human study

**DOI:** 10.1007/s10637-022-01247-1

**Published:** 2022-04-25

**Authors:** Patricia LoRusso, Mark J. Ratain, Toshihiko Doi, Drew W. Rasco, Maja J. A. de Jonge, Victor Moreno, Benedito A. Carneiro, Lot A. Devriese, Adam Petrich, Dimple Modi, Susan Morgan-Lappe, Silpa Nuthalapati, Monica Motwani, Martin Dunbar, Jaimee Glasgow, Bruno C. Medeiros, Emiliano Calvo

**Affiliations:** 1 Yale Cancer Center, New Haven, CT USA; 2 University of Chicago, Chicago, IL USA; 3 National Cancer Center Hospital East, Kashiwa, Japan; 4 START, San Antonio, TX USA; 5 Erasmus MC Cancer Institute, Rotterdam, The Netherlands; 6 START Madrid-FJD, Hospital Fundación Jiménez Díaz, Madrid, Spain; 7 Lifespan Cancer Institute, Cancer Center at Brown University, Providence, RI USA; 8 Department of Medical Oncology, University Medical Center Utrecht, Utrecht, The Netherlands; 9 AbbVie Inc, North Chicago, IL USA; 10 START Madrid-CIOCC, Centro Integral Oncológico Clara Campal, Madrid, Spain

**Keywords:** TRAIL-R agonist, Solid tumor, Dose-escalation, Dose-optimization, Phase 1, Apoptosis

## Abstract

Eftozanermin alfa (eftoza), a second-generation tumor necrosis factor-related apoptosis-inducing ligand receptor (TRAIL-R) agonist, induces apoptosis in tumor cells by activation of death receptors 4/5. This phase 1 dose-escalation/dose-optimization study evaluated the safety, pharmacokinetics, pharmacodynamics, and preliminary activity of eftoza in patients with advanced solid tumors. Patients received eftoza 2.5–15 mg/kg intravenously on day 1 or day 1/day 8 every 21 days in the dose-escalation phase, and 1.25–7.5 mg/kg once-weekly (QW) in the dose-optimization phase. Dose-limiting toxicities (DLTs) were evaluated during the first treatment cycle to determine the maximum tolerated dose (MTD) and recommended phase 2 dose (RP2D). Pharmacodynamic effects were evaluated in circulation and tumor tissue. A total of 105 patients were enrolled in the study (dose-escalation cohort, n = 57; dose-optimization cohort, n = 48 patients [n = 24, colorectal cancer (CRC); n = 24, pancreatic cancer (PaCA)]). In the dose-escalation cohort, seven patients experienced DLTs. MTD and RP2D were not determined. Most common treatment-related adverse events were increased alanine aminotransferase and aspartate aminotransferase levels, nausea, and fatigue. The one treatment-related death occurred due to respiratory failure. In the dose-optimization cohort, three patients (CRC, n = 2; PaCA, n = 1) had a partial response. Target engagement with regard to receptor saturation, and downstream apoptotic pathway activation in circulation and tumor were observed. Eftoza had acceptable safety, evidence of pharmacodynamic effects, and preliminary anticancer activity. The 7.5-mg/kg QW regimen was selected for future studies on the basis of safety findings, pharmacodynamic effects, and biomarker modulations. (*Trial registration number*: NCT03082209 (registered: March 17, 2017)).

**Supplementary Information:**

The online version contains supplementary material available at 10.1007/s10637-022-01247-1.

## Introduction

Tumor necrosis factor (TNF)-related apoptosis-inducing ligand (TRAIL) is a member of the TNF superfamily of proteins that help activate intracellular signaling pathways involved in cell proliferation, survival, and apoptosis [1]. The ability of TRAIL to selectively induce the extrinsic apoptotic pathway in cancer cells via its trimeric binding to cell surface death receptors TRAIL-R1 (DR4) and TRAIL-R2 (DR5) provided the basis for developing TRAIL receptor agonists as a therapeutic approach [1, 2]. Although activation of the TRAIL pathway has emerged as an attractive therapeutic strategy in cancer, and early phase 1 trials yielded encouraging preliminary data, this did not translate to significant clinical benefit in subsequent phase 2 studies [2, 3]. The limited efficacy of first-generation TRAIL receptor agonists likely reflects weak agonistic activity and several resistance mechanisms through which tumor cells escape TRAIL-induced apoptosis [1, 2, 4]. Some of these factors include reduced expression of DRs, upregulation of anti-apoptotic proteins, and suppression of the caspase cascade [5–7]. However, the major contributing factor to the lack of clinical activity by these first-generation agonists has been ascribed to suboptimal TRAIL receptor clustering that in some cases requires additional Fc-FcγR–mediated cross-linking [8–10].

Eftozanermin alfa (formerly known as ABBV-621) is a second-generation TRAIL receptor agonist comprising a human immunoglobulin G1-Fc mutant backbone linked to two sets of trimeric native single-chain TRAIL receptor-binding domain monomers that bind to death-inducing DR4 and DR5 [4, 11]. Eftozanermin alfa thus contains a total of six DR-binding sites per molecule to maximize optimal receptor clustering on tumor cells. In human hematologic and solid tumor cell lines, eftozanermin alfa selectively binds to DR4 and DR5 with nanomolar affinity to drive on-target biologic activity with enhanced caspase-8 aggregation, death-inducing signaling complex formation, and potent caspase-dependent antitumorigenic activity that is independent of Fc-FcγR interactions [4, 12]. Using an in vivo patient-derived xenograft (PDX) screen across 15 different tumor indications, eftozanermin alfa demonstrated a range of potent antitumor activity, with PDX response rates of 45% (10/22) in colorectal cancer (CRC) and 35% (7/20) in pancreatic cancer (PaCA) [4]. In addition, activity was found to be significantly enhanced when combined with chemotherapeutics (e.g., taxanes or topoisomerase-1 inhibitors) or inhibitors of B-cell lymphoma-extra-large protein [4].

This first-in-human, open-label, phase 1 study aimed to determine the maximum tolerated dose (MTD), recommended phase 2 dose (RP2D), safety, pharmacokinetics, pharmacodynamics, and antitumor activity of eftozanermin alfa as monotherapy in patients with advanced solid tumors and hematologic malignancies. This manuscript reports results from the eftozanermin alfa monotherapy dose-escalation and dose-optimization cohorts in patients with solid tumors.

## Methods

### Study design

The primary objectives of this phase 1, first-in-human, open-label, multicenter study (NCT03082209) were to establish the MTD/RP2D of eftozanermin alfa, and to determine its pharmacokinetic profile. Secondary objectives were to assess safety and tolerability of the compound and define dose-limiting toxicities (DLTs). Exploratory objectives included evaluation of preliminary antitumor efficacy, and assessment of the pharmacodynamic effect and association with pharmacokinetics, safety, and efficacy. The study consisted of two cohorts: dose escalation and dose optimization. Patients in both cohorts underwent screening procedures within 21 days before eftozanermin alfa administration.

### Dose-escalation/dose-optimization cohorts

Patients with solid tumors received intravenous infusions of eftozanermin alfa at a starting dose of 2.5 mg/kg administered on day (D)1 of a 21-day cycle (D1 every 3 weeks [Q3W]). Once the criteria for dose escalation were met, the second dose level of 2.5 mg/kg administered on D1 and 8 of a 21-day cycle (D1/D8 Q3W) was initiated, and subsequently, doses were escalated (2.5, 3.75, 5, 6.5, 8.5, 11, and 15 mg/kg) with the frequency of D1/D8 Q3W. Dose escalation was guided by a Bayesian continual reassessment method (to determine the MTD) not exceeding a two-fold increase in total dose administered over the 21-day cycle. The dose-optimization cohort was initiated to inform the RP2D. Three concurrent, non-randomized dose levels were explored with eftozanermin alfa (1.25, 3.75, 7.5 mg/kg) administered intravenously as monotherapy on a once-weekly (QW) dosing schedule (D1, 8, and 15 of a 21-day cycle) in patients with *KRAS*-mutated CRC and in patients with PaCA. Although there is high prevalence of *KRAS* mutation in PaCA (> 90%), confirmation of *KRAS* status was not a criterion for enrollment.

The study was conducted in accordance with International Conference on Harmonization Good Clinical Practice guidelines and the study protocol was approved by an independent ethics committee/institutional review board at each participating site. All patients provided written informed consent.

### Patients

Patients aged ≥ 18 years with Eastern Cooperative Oncology Group performance status 0–2 and diagnosed with a solid tumor with measurable disease by Response Evaluation Criteria in Solid Tumors (RECIST) v1.1 were eligible. Patients with non-Hodgkin lymphoma with measurable disease per Lugano classification were eligible for the dose-escalation cohort, but none were enrolled. Patients in the dose-optimization cohort included those with *KRAS-*mutated CRC or PaCA (irrespective of mutational status). Patients in both cohorts were additionally required to have had prior treatment with one or more systemic therapy and relapsed/progressed on available standard therapies. All eligible patients needed adequate hematologic, renal, and hepatic function, and to consent to providing tumor tissue (archived or fresh tumor) for biomarker assessment. In addition, at least 24 patients from dose-optimization cohorts had to consent to two fresh biopsies (pre-treatment and on-treatment) for pharmacodynamic assessment (Online Resource 1 – cohort diagram). The latter portion of the study was limited to those sites agreeable to performing the mandatory biopsies.

Patients meeting any of the following criteria were excluded from the study: presence of primary hepatobiliary malignancy (criterion added after dose level 4); undergone major surgery within 4 weeks of first dose of study drug; received any systemic anticancer treatment within 21 days or three half-lives prior to first dose of study drug (whichever was longer); history of brain metastases without clinical/radiographic stable disease (SD) for ≥ 28 days after definitive therapy; or any clinically significant, uncontrolled medical condition.

### Assessments

Routine safety evaluations were performed and adverse-event (AE) severity was assessed by the investigator via the National Cancer Institute Common Terminology Criteria for Adverse Events v4.03.

The DLT assessment period was defined as beginning on the first day of eftozanermin alfa dosing and continuing for 21 days. DLTs for dose escalation and dose optimization were determined during the first cycle and were defined as: any eftozanermin alfa-related grade ≥ 3 toxicity; grade 3 mucositis, nausea, vomiting, or diarrhea lasting > 72 h, or grade 4 of any duration; > 7-consecutive-day treatment delay due to eftozanermin alfa-related toxicity; grade 4 clinically significant laboratory non-hematologic toxicity lasting > 24 h; any grade 4 hematologic toxicity; or any AE meeting the definition of Hy’s law.

Pharmacokinetic sampling details are presented in Online Resource 1. Eftozanermin alfa serum concentrations were determined using a validated electrochemiluminescence immunoassay (Online Resource 1).

Tumor assessments were performed at baseline within 28 days of cycle (C)1D1, and post-baseline within 7 days before dosing on C3D1, and Q9W thereafter. Tumor response was evaluated using RECIST v1.1. Tumor assessments were made per local investigators for all patients and required confirmation by central radiologists for the CRC dose-optimization cohort.

### Pharmacodynamic and exploratory biomarker assessments

Pharmacodynamic and biomarker analyses were conducted to demonstrate receptor binding of eftozanermin alfa with TRAIL receptors, and confirm apoptotic pathway activation in tumor; details are presented in Online Resource 1. Twenty-five patients from the dose-optimization cohort (CRC and PaCA) consented to fresh pre- and on-treatment (paired) tumor biopsies (Online Resource 1 – cohort diagram). Pre-treatment biopsies were collected anytime during the screening period – within 28 days before starting the study treatment – and on-treatment biopsies were collected 24 ± 4 h following second or third infusion. Formalin-fixed paraffin-embedded (FFPE) and flash-frozen core biopsy samples were collected. The FFPE paired tumor tissues were used to assess apoptotic pathway activation (cleaved poly[ADP-ribose] polymerase [c-PARP]) by immunohistochemistry (IHC) and immune infiltrate (by multiplex immunofluorescence and RNA whole‐transcriptome sequencing assays) (Online Resource 1). The frozen paired tumor tissues were used for reverse-phase protein array (RPPA) analysis (George Mason University, Manassas, VA) (Online Resource 1).

DR4 IHC was performed on archival and fresh biopsy if available on FFPE tissue, at CellCarta (formerly HistoGeneX, Antwerp, Belgium) using rabbit monoclonal antibody clone D9S1R, Cell Signaling #42,533 (Danvers, MA/USA). Staining was performed on the Leica BOND RX instrument (Buffalo Grove, IL, USA). Staining on membrane, cytoplasmic, and overall were separately scored. Scoring included intensity of staining and proportion of cells staining at a certain intensity (Online Resource 2 – DR4/5 levels).

### Statistical analyses

AE analyses were based on treatment-emergent AEs (TEAEs), i.e., those AEs with onset on/after the day of first dose of study drug and no more than 30 days after last dose of study drug.

Efficacy was assessed through objective response rate (defined as confirmed complete response [CR] or partial response [PR]), best response, and disease control rate (defined as CR, PR, or SD ≥ 5 weeks away from the date of first dose of study drug).

Statistical analyses for pharmacodynamic assessments are described in Online Resource 1.

## Results

### Demographics and baseline characteristics

Between March 29, 2017 and February 14, 2018, 57 patients were enrolled in the dose-escalation cohort. Between March 28, 2018 and February 11, 2019, 24 patients with CRC and 24 with PaCA were enrolled in the dose-optimization cohort. Baseline demographics and clinical characteristics of both cohorts are summarized in Table 1.

**Table 1:** Patient demographics and baseline characteristics

**Characteristic**	**Dose escalation**	**Dose optimization**		
	**(N = 57)**	**CRC**	**PaCA**	**Total**
		**(n = 24)**	**(n = 24)**	**(N = 48)**
Age, median (range), years	61 (34–82)	60 (43–76)	65 (48–76)	63 (43–76)
Sex, n (%)				
Male	34 (60)	15 (63)	16 (67)	31 (65)
Female	23 (40)	9 (38)	8 (33)	17 (35)
Race, n (%)				
White	42 (74)	22 (92)	22 (92)	44 (92)
Black	4 (7)	1 (4)	1 (4)	2 (4)
Asian	11 (19)	1 (4)	1 (4)	2 (4)
Primary tumor type, n (%)				
Colorectal cancer	13 (23)	24 (100)	0	24 (50)
Pancreatic cancer	17 (30)	0	24 (100)	24 (50)
Sarcoma	4 (7)	0	0	0
Bile duct cancer^a^	3 (5)	0	0	0
Other solid tumors	20 (35)	0	0	0
No. of prior treatments, median (range)	4 (1–10)	4 (2–8)	3 (1–7)	3 (1–8)
*KRAS* mutation status, n (%)				
Mutated	13 (23)	24 (100)	8 (33)	32 (67)
Unmutated	15 (26)	0	1 (4)	1 (2)
Missing	29 (51)	0	15 (63)	15 (31)

^a^Bile duct cancer/cholangiocarcinoma patients enrolled in dose levels 1 to 4 only; after dose level 4 (5 mg/kg D1/D8 Q3W), a protocol amendment excluded patients with primary hepatobiliary malignancy due to increased risk for hepatic lab abnormalities during eftozanermin alfa treatment

*CRC* colorectal cancer, *D* day, *PaCA* pancreatic cancer, *Q3W* every 3 weeks

### Exposure and disposition

As of the March 6, 2020 data cutoff, all 57 patients in the dose-escalation cohort received one or more treatment cycles, with a median two treatment cycles (range, 1–11). Median treatment duration was 29 days (range, 1–221). The most common reason for eftozanermin alfa discontinuation was disease progression (n = 40; 70%), followed by AEs (n = 7; 12%) and consent withdrawal (n = 3; 5%). Two patients discontinued eftozanermin alfa due to physician’s decision (4%), and five other patients discontinued study drug due to other reasons (9%). In the dose-optimization cohorts, all 48 patients received one or more doses of eftozanermin alfa, with a median two treatment cycles (range, 1–14). Median treatment duration was 36 days (range, 1–290). Patient disposition for the dose-optimization cohort is summarized in Online Resource 3 – Fig. S1. Thirty-eight (79%) patients discontinued eftozanermin alfa due to progressive disease.

### Safety

In the dose-escalation cohort, 54 of 57 patients completed all scheduled doses of eftozanermin alfa during the 21-day DLT evaluation period. The MTD was not identified, inclusive of the highest tested dose of 15 mg/kg. In total, seven patients experienced DLTs (Online Resource 3 – Table S1). Fifty-five (97%) patients experienced TEAEs, with 31 (54%) patients experiencing grade ≥ 3 TEAEs, and 22 (39%) patients reporting serious AEs (SAEs). Frequently reported TEAEs were fatigue (39%), nausea and tumor pain (35% each), decreased appetite and pyrexia (26% each), diarrhea (25%), vomiting (23%), and alanine aminotransferase (ALT) increase (21%). AEs were assessed as treatment related in 43 (75%) patients; the most common are summarized in Table 2.

**Table 2:** Summary of treatment-related adverse events occurring in ≥ 15% of patients in either the dose-escalation or dose-optimization cohorts of the study

**Treatment-related adverse event, n (%)**	**Dose escalation**		**Dose optimization**					
	**(N = 57)**		**CRC (n = 24)**		**PaCA (n = 24)**		**Total (N = 48)**	
	**Any TRAE**	**Grade ≥ 3 TRAE**	**Any TRAE**	**Grade ≥ 3 TRAE**	**Any TRAE**	**Grade ≥ 3 TRAE**	**Any TRAE**	**Grade ≥ 3 TRAE**
Patients with ≥ 1 TRAE	43 (75)	12 (21)	22 (92)	4 (17)	22 (92)	6 (25)	44 (92)	10 (21)
Increased ALT	11 (19)	7 (12)	4 (17)	1 (4)	6 (25)	2 (8)	10 (21)	3 (6)
Increased AST	10 (18)	5 (9)	4 (17)	2 (8)	4 (17)	2 (8)	8 (17)	4 (8)
Nausea	10 (18)	1 (2)	2 (8)	0	6 (25)	0	8 (17)	0
Diarrhea	7 (12)	1 (2)	1 (4)	0	6 (25)	0	7 (15)	0
Stomatitis	7 (12)	0	5 (21)	0	4 (17)	0	9 (19)	0
Fatigue	5 (9)	1 (2)	7 (29)	0	4 (17)	0	11 (23)	0
Vomiting	5 (9)	0	1 (4)	0	6 (25)	0	7 (15)	0
Decreased appetite	5 (9)	0	4 (17)	0	3 (13)	0	7 (15)	0

*ALT* alanine aminotransferase, *AST* aspartate aminotransferase, *CRC* colorectal cancer, *PaCA* pancreatic cancer, *TRAE* treatment-related adverse event

In the dose-optimization cohort, all patients received at least one dose of eftozanermin alfa. Three (6%) patients experienced treatment-related AEs (TRAEs; not considered DLTs) that led to study discontinuation, including increases in ALT, aspartate aminotransferase (AST), and/or bilirubin, and respiratory failure in one patient with PaCA receiving 7.5 mg/kg. Four (8%) patients reported DLTs possibly associated with eftozanermin alfa treatment (Online Resource 3 – Table S1). All patients experienced one or more TEAEs regardless of attribution to study treatment, including 31 (65%) patients with grade ≥ 3 TEAEs and 27 (56%) patients with SAEs. There was no apparent relationship of grade ≥ 3 TEAEs to eftozanermin alfa dose (Online Resource 3 – Table S2). Most frequently reported TEAEs were fatigue (46%), constipation (35%), diarrhea (29%), nausea (27%), increased ALT and AST (25% each), decreased appetite and vomiting (23% each), and stomatitis (21%). TRAEs occurred in 44 (92%) patients, including 10 (21%) with grade ≥ 3 TRAEs and seven (15%) with treatment-related SAEs. The most common TRAEs were hepatic or constitutional (Table 2). Sixteen patients received the 7.5-mg/kg QW regimen; all reported TRAEs, including five (31%) with grade ≥ 3 TRAEs and three (19%) with treatment-related SAEs (Online Resource 3 – Table S2). The most common TRAEs at the 7.5-mg/kg QW dose were gastrointestinal disorders, with stomatitis (n = 5, 31%) and nausea (n = 4, 25%) the most frequently reported events.

### Pharmacokinetics

**Fig. 1:**
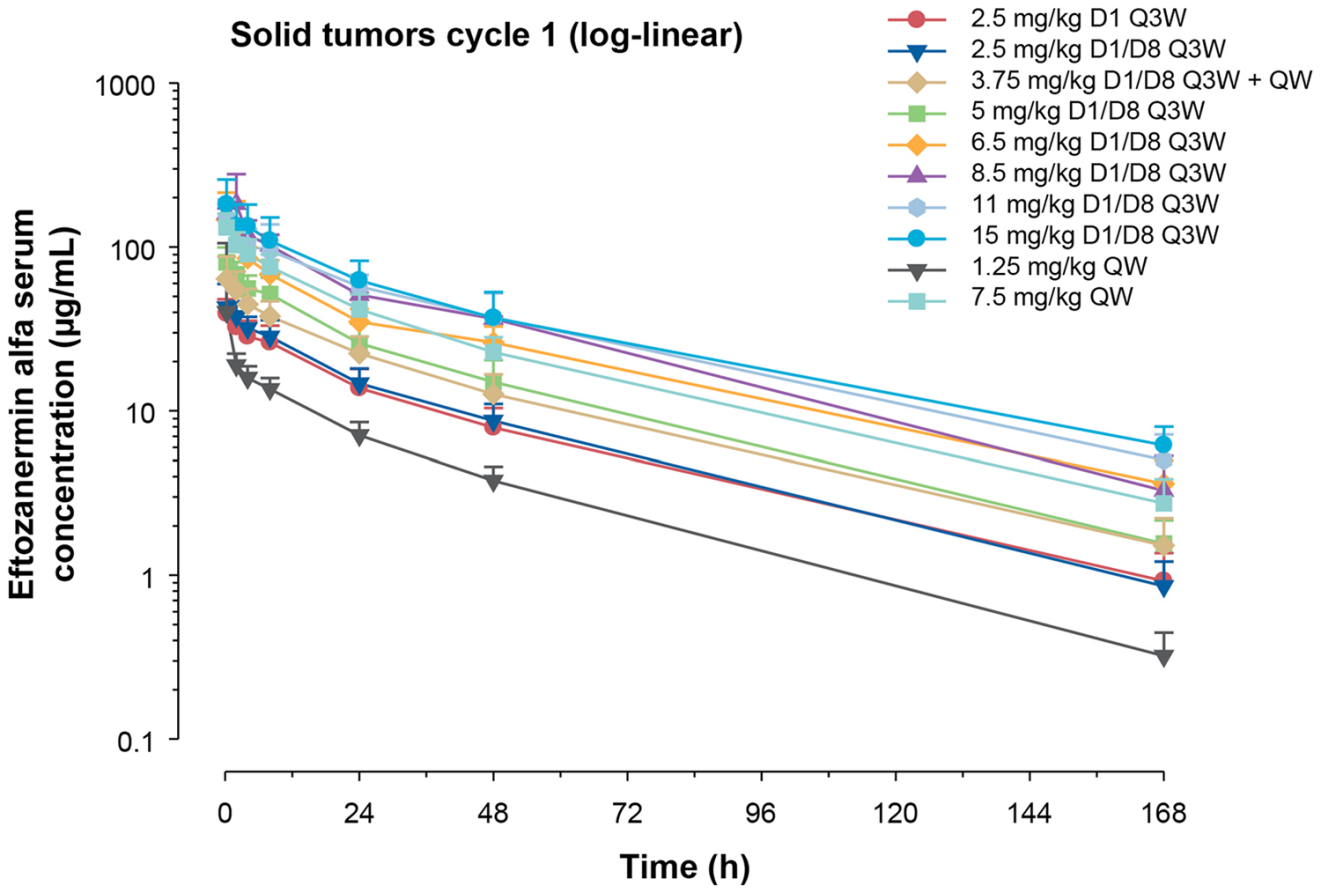
Mean (SD) log-linear plasma concentration–time profiles of eftozanermin alfa in dose-escalation and dose-optimization cohorts. D1, day 1; D8, day 8; Q3W, every 3 weeks; QW, once-weekly; SD, standard deviation

In the dose-escalation cohort, after D1 and D1/D8 intravenous infusion at doses of 2.5–15 mg/kg Q3W, the mean systemic exposure of eftozanermin alfa showed dose-proportional kinetics in the dose range of 1.25–7.5 mg/kg weekly (Fig. 1). Pharmacokinetics were also dose proportional in the dose-optimization cohort after single-dose administration of eftozanermin alfa on C1D1 in the dose range of 1.25–7.5 mg/kg (Fig. 1; Online Resource 3 – Table S3). The half-life of eftozanermin alfa ranged from 21–46 h. No accumulation after multiple doses was observed.

### Efficacy

**Fig. 2:**
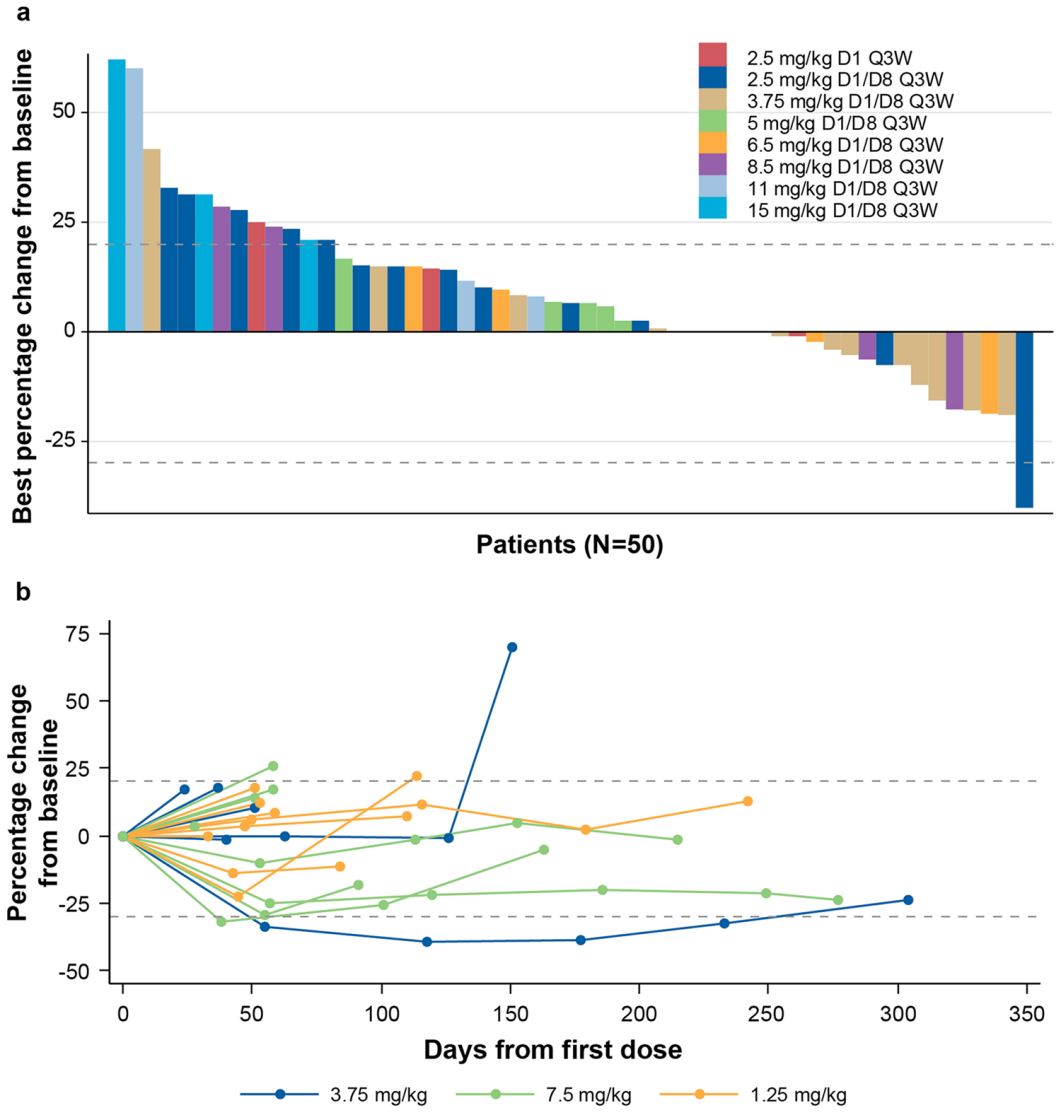
Efficacy evaluation of eftozanermin alfa: (**a**) Best percentage change in size of target lesions from baseline in patients with one or more post-baseline tumor assessment (dose-escalation cohort); (**b**) Spider plot of percentage change in size of tumor lesions over time in patients with colorectal cancer with one or more post-baseline tumor assessment (dose-optimization cohort). Baseline tumor assessments were performed at D1 (baseline), within 28 days of C1D1, within 7 days before dosing on C3D1 (post-baseline), and Q9W thereafter. C, cycle; D, day; Q3W, every 3 weeks; Q9W, every 9 weeks

In the dose-escalation cohort, 50 patients had one or more post-baseline tumor assessments (Fig. 2a). Best responses to eftozanermin alfa monotherapy included a PR lasting 11 weeks in a patient with PaCA in the 2.5-mg/kg D1/D8 cohort and SD lasting > 12 weeks in six patients. In the dose-optimization cohort, a confirmed PR was observed in two patients with CRC (central read; 7.5 mg/kg) and one patient with PaCA (local read; 1.25 mg/kg). In addition, 20 (42%) patients achieved a best response of SD (local read) at an average of 10 weeks of treatment with eftozanermin alfa monotherapy, including nine (38%) patients with CRC and 11 (46%) with PaCA. Figure 2b and Online Resource 3 – Fig. S2 show the percentage change in size of tumor lesions over time for each patient with CRC or PaCA and one or more post-baseline tumor assessments (dose-optimization cohort).

### Pharmacodynamic and exploratory biomarker analyses

**Fig. 3:**
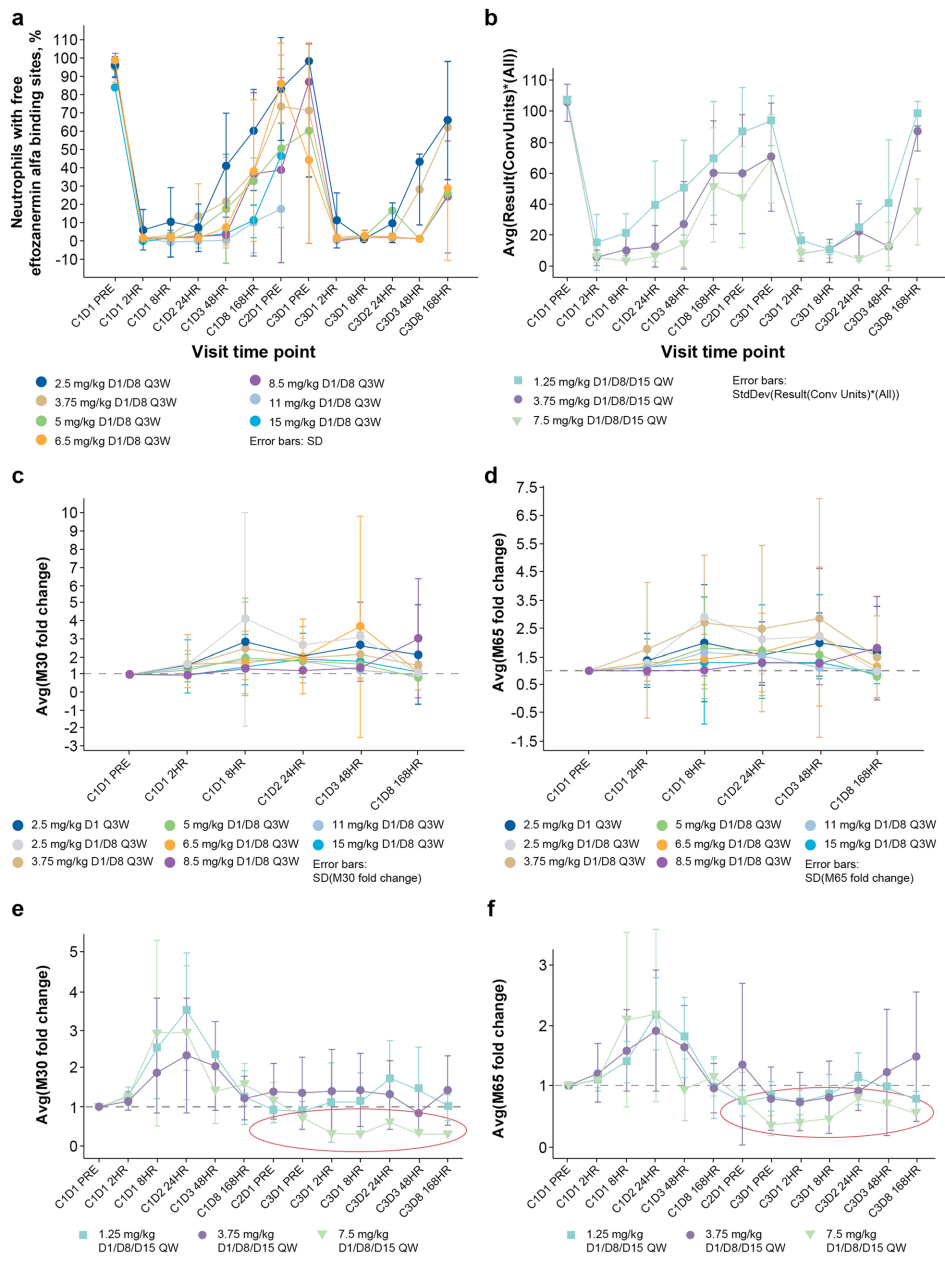
Biomarker analysis by dose level following eftozanermin alfa administration in the dose-escalation (**a**, **c**, **d**) and dose-optimization (**b**, **e**, **f**) cohorts. TRAIL receptor occupancy (**a**); saturation of eftozanermin alfa binding sites (**b**); mean fold change (± SD) in serum M30 (**c**) and M65 (**d**) levels; mean fold change (± SD) in serum M30 (**e**) and M65 (**f**) levels in patients with colorectal cancer. **C**, cycle; **D**, day; HR, hour; PRE, pre-dose; Q3W, every 3 weeks; QW, once-weekly; SD, standard deviation; TRAIL, tumor necrosis factor-related apoptosis-inducing ligand

In the dose-escalation cohort, complete saturation of all eftozanermin alfa-binding sites (both decoy receptor 1 and 2) of the TRAIL receptor occurred in blood neutrophils at 2 h post-dosing for all doses evaluated (Fig. 3a). This was followed by gradual receptor desaturation across the dose range, returning to near pre-dose levels before the start of the next eftozanermin alfa dosing cycle. These changes were confirmed in the dose-optimization cohort, with complete saturation of all eftozanermin alfa-binding sites on neutrophils 2 h after dosing at all doses evaluated, followed by dose-dependent time to receptor desaturation (Fig. 3b). Since TRAIL receptors are also expressed on epithelial cells of normal tissues, appearance of the circulating apoptosis markers M30 and M65 that are shed in the bloodstream following cell death in epithelial cells was also assessed, to demonstrate target engagement. Increases in serum levels of M30 and M65 were observed at 8, 24, and 48 h post-dose, with these changes occurring independently of dose (Fig. 3c, d). Transient increases in both M30 and M65 levels were observed with all doses tested in patients with CRC (Fig. 3e, f), and also in patients with PaCA (Online Resource 3 – Fig. S3). Furthermore, M65 and M30 were consistently lower than baseline levels after C2 in patients with CRC who received the 7.5-mg/kg dose.

Overall, tumor biomarker data for exploratory analyses were from a small number of patients, which limited the power of these analyses. Of the twenty-five patients who consented to provide fresh biopsies, evaluable FFPE tissue was available on 11 paired pre- and on-treatment biopsies. Biopsies from the other 14 patients were not evaluable, either because the pre- or on-treatment samples lacked tumor cells, or because on-treatment samples were not collected (patients withdrew consent or investigator determined that biopsy may not be safe). Eight of 11 pairs were assessed for c-PARP levels by IHC (three pairs were non-evaluable due to limited tissue or assay failure). Seven of eight on-treatment biopsies showed increased levels of c-PARP relative to pre-treatment biopsies (Online Resource 2 – Fig. S1c, S1d).

Tumor biopsies that were collected as frozen cores were available for four pairs. Analysis of frozen paired biopsies by RPPA showed two- to four-fold increase in c-PARP in three of four on-treatment biopsies compared with pre-treatment tumor tissue (Online Resource 2 – Fig. S1a, S1b). Additionally, levels of pro-survival pathway molecules such as p-MEK1/2, p-AKT, and p-ERK decreased in three of four on-treatment tumor tissue samples compared with pre-treatment tumor tissue. Parenthetically, as per protocol the clinical sites prioritized the first cores for formalin fixation; hence, the majority of frozen cores had very low/no tumor content. Data on RPPA are described in Online Resource 2 – Fig. S1.

Tumor-infiltrating immune cells were also assessed using RNA sequencing data from FFPE tumor tissues. Of 11 pairs, six had on-treatment biopsies with two- to three-fold higher levels of total immune cells relative to pre-treatment biopsies. The CD4-positive (CD4 +) naive T cells were also higher in seven of 11 on-treatment biopsies relative to pre-treatment biopsies. The immunofluorescence assay also showed increase in CD4 + cells in on-treatment biopsies compared with pre-treatment biopsy in the majority of paired samples (Online Resource 2 – Fig. S2). The small cohort size limits robust statistical analysis, and as such these data demonstrate the trends of increased immune infiltrate in tumor following eftozanermin alfa treatment.

Additional biomarker data on DR4/5 levels in archival or fresh tumor tissues are reported in Online Resource 2.

## Discussion

This first-in-human phase 1 study showed that administration of eftozanermin alfa monotherapy in patients with previously treated solid tumors was well tolerated, with the drug showing an acceptable safety profile at all dose levels. The MTD was not identified in the dose-escalation cohort, in which eftozanermin alfa was administered at doses of up to 15 mg/kg in a D1/D8 Q3W schedule. Although all doses were tolerated, dose-proportional kinetics were only observed in the dose range of 1.25–7.5 mg/kg weekly. Subsequent evaluation of eftozanermin alfa at three concurrent (non-randomized) dose levels up to 7.5 mg/kg QW in dose-optimization cohorts that included patients with PaCA and *KRAS*-mutated CRC confirmed the safety findings of the dose-escalation cohort, and permitted an expanded evaluation of eftozanermin alfa safety, pharmacokinetics, and pharmacodynamics. The most common AEs related to treatment with eftozanermin alfa were increased ALT and AST levels, nausea, and fatigue. The safety findings of eftozanermin alfa in the present study were broadly consistent with other TRAIL receptor agonists [13, 14], including the safety profiles of DR5 agonist DS-8273a, and dual Apo2L/TRAIL agonist [15, 16]. Eftozanermin alfa showed dose-proportional pharmacokinetics, with a half-life ranging from 21–46 h. Evidence of antitumor efficacy was also observed, with one patient with PaCA in the dose-escalation cohort and three patients in the dose-optimization cohort (CRC, n = 2; PaCA, n = 1) achieving PR. On the basis of the collective safety/tolerability, efficacy, and pharmacokinetic and pharmacodynamic data, 7.5 mg/kg QW was selected (due to lack of additional increase in exposure beyond this dose level) as the dose for further evaluation in ongoing studies.

The present study confirms the pharmacodynamic effects of eftozanermin alfa treatment through saturation of TRAIL receptor occupancy and subsequent engagement of the apoptotic pathway. The longitudinal quantitation of circulating full-length (M65) and caspase-cleaved (M30) cytokeratin 18 in serum showed that cells were undergoing apoptosis following eftozanermin alfa treatment. The dose-dependent time to receptor desaturation was observed with receptor occupancy assay; however, increase in M65 and M30 serum concentrations or in tumor were not dose dependent. M65 and M30 were consistently lower than baseline levels after C2 in patients with CRC who received the 7.5-mg/kg dose, suggestive of a potential decrease in tumor burden.

Several exploratory biomarker analyses demonstrated apoptotic cell death and impact on infiltrating tumor cells following eftozanermin alfa in paired fresh pre- and on-treatment tumor tissues. Although tumor biomarker data from small numbers of patients limited the power of these analyses, we observed increased PARP cleavage in tumor cells following eftozanermin alfa dosing, demonstrating activation of the agent’s key mechanism of action. In addition, decreased cell proliferation/survival signaling pathways (MEK/Erk/AKT), and potentially increased tumor-infiltrating lymphocytes in tumor tissue after eftozanermin alfa dosing, were also observed. The mechanism of decrease in MEK/AKT signaling by eftozanermin alfa is not fully understood. Incidentally, these pathways are active in *KRAS*-mutated tumors, and a decrease in these pathways may be partially due to cell killing by eftozanermin alfa.

The impact of TRAIL receptor agonists on immune cells, notably depletion/decrease in myeloid-derived suppressor cells, was reported in mice and humans previously [17, 18]. We observed a trend toward increase in CD4 + T cells in tumor following eftozanermin alfa treatment. Mandatory biopsies, albeit with low numbers, have provided preliminary evidence on pharmacodynamic effect in tumor following eftozanermin alfa dosing. The sponsor has adopted American Society of Clinical Oncology guidelines regarding these procedures [19].

Association between clinical responses and target expression could not be established due to limited data. As such, we observed higher DR4 and DR5 gene expression in pre-treatment fresh tumor biopsy specimens compared with archival tissue. Although this observation suggests the prior lines of treatment result in DR4/5 expression increase, other factors such as heterogeneity in different lesions/anatomic sites of biopsies, and the age of tumor samples cannot be ruled out (data summarized in Online Resource 3 [figures]). In light of this observation, it is advisable in future studies to evaluate fresh biopsies for DR4/5 assessments, to establish the association between target expression and eftozanermin alfa activity.

In conclusion, the most common TRAEs associated with eftozanermin alfa administration were liver related or constitutional, and manageable. At the selected 7.5-mg/kg QW regimen dose exposure, eftozanermin alfa had acceptable tolerability and safety while achieving more-frequent relevant tumor regressions in CRC. The reported study provides a foundation for trials in patients with CRC assessing therapy with eftozanermin alfa, as monotherapy or in combinations to overcome resistance mechanisms.

## Supplementary Information

Below is the link to the electronic supplementary material.

Supplementary file1 (DOCX 153 KB)

Supplementary file2 (PDF 844 KB)

Supplementary file3 (PDF 464 KB)

## Data Availability

AbbVie is committed to responsible data sharing regarding the clinical trials we sponsor. This includes access to anonymized, individual and trial-level data (analysis data sets), as well as other information (e.g., protocols and Clinical Study Reports), as long as the trials are not part of an ongoing or planned regulatory submission. This includes requests for clinical trial data for unlicensed products and indications. These clinical trial data can be requested by any qualified researchers who engage in rigorous, independent scientific research, and will be provided following review and approval of a research proposal and Statistical Analysis Plan (SAP) and execution of a Data Sharing Agreement (DSA). Data requests can be submitted at any time and the data will be accessible for 12 months, with possible extensions considered. For more information on the process, or to submit a request, visit the following link: https://www.abbvie.com/our-science/clinical-trials/clinical-trials-data-and-information-sharing/data-and-information-sharing-with-qualified-researchers.html.
