# Supplementary material for: Eftozanermin alfa (ABBV-621) monotherapy in patients with previously treated solid tumors: findings of a phase 1, first-in-human study
Source: Invest New Drugs. 2022 Apr 25;40(4):762–72. doi: 10.1007/s10637-022-01247-1 (PMC9035501; doi:10.1007/s10637-022-01247-1)
Supplement: Supplementary file 1 — Supplementary file1 (DOCX 153 KB) [file 10637_2022_1247_MOESM1_ESM.docx]

**Eftozanermin alfa (ABBV-621) monotherapy in patients with previously treated solid tumors: findings of a phase 1, first-in-human study**

Patricia LoRusso^1^, Mark J. Ratain^2^, Toshihiko Doi^3^, Drew W. Rasco^4^, Maja J.A. de Jonge^5^, Victor Moreno^6^, Benedito A. Carneiro^7^, Lot A. Devriese^8^, Adam Petrich^9,*^, Dimple Modi^9^, Susan Morgan-Lappe^9^, Silpa Nuthalapati^9,*^, Monica Motwani^9^, Martin Dunbar^9^, Jaimee Glasgow^9^, Bruno C. Medeiros^9^, Emiliano Calvo^10^

^1^Yale Cancer Center, New Haven, CT, USA; ^2^University of Chicago, Chicago, IL, USA; ^3^National Cancer Center Hospital East, Kashiwa, Japan; ^4^START, San Antonio, TX, USA; ^5^Erasmus MC Cancer Institute, Rotterdam, The Netherlands; ^6^START Madrid-FJD, Hospital Fundación Jiménez Díaz, Madrid, Spain; ^7^Cancer Center at Brown University, Lifespan Cancer Institute, Providence, RI, USA; ^8^Department of Medical Oncology, University Medical Center Utrecht, Utrecht, The Netherlands; ^9^AbbVie Inc., North Chicago, IL, USA; ^10^START Madrid-CIOCC, Centro Integral Oncológico Clara Campal, Madrid, Spain

*Former employee of AbbVie.

**Corresponding author:**

Emiliano Calvo, MD, PhD

START Madrid-CIOCC

Centro Integral Oncológico Clara Campal

Hospital Madrid Norte Sanchinarro

Calle Oña, 10. 28050 Madrid, Spain

Email: emiliano.calvo@startmadrid.com

**Target journal:** Investigational New Drugs

**Online Resource 1 – Methods**

Pharmacokinetic analysis

Blood samples for pharmacokinetic analysis were collected in cycle (C)1 and 3 pre-dose, and 0.25, 2, 4, 8, 24, 48, 168, and 336 h post-dose; in C2 pre-dose, and 0.25, 24, and 168 h post-dose; C4–16 on day 1 pre-dose; after C16, Q8W pre-dose; after C36, Q16W pre-dose; and at the final visit. Pharmacokinetic parameters, including maximum observed plasma concentration (C_max_), the time to C_max_, and area under the plasma concentration-time curve were calculated using non-compartmental methods for the dose-optimization cohort.

Eftozanermin alfa quantification

The method for the determination of eftozanermin alfa in human serum was based on an electrochemiluminescence ligand-binding assay in a "step-by-step" format.

For the quantification of eftozanermin alfa, streptavidin-coated electrochemiluminescent (ECL) 96-well standard plates (Meso Scale Discovery, Rockville, MD) were used. After a blocking step (incubation of 1×tris-buffered saline [TBS]/1% casein for 1 hour), plates were washed and subsequently incubated with Bio Anti Id mAb 2 ABBV-621, which binds to the streptavidin coating of the ECL 96-well plate. Unbound material was removed by washing. Standards (STDs) and quality control (QC) samples were diluted 1:10 using 0.1×TBS/0.1% casein buffer. Unknown study samples were also diluted 1:10 or higher. The diluted STDs, QCs, and study samples were transferred to the pre-treated ECL 96-well plate and incubated for 1 hour. Eftozanermin alfa bound to Bio Anti Id mAb 2 eftozanermin alfa; unbound material was removed by washing. Subsequently, plates were incubated with Sulfo Anti Id mAb eftozanermin alfa for 1 hour to enable detection of captured eftozanermin alfa molecules. After a subsequent washing step to remove unbound material, ECL plates were incubated using MSD read buffer two times for 5 minutes and emitted light signals were detected using a QuickPlex MSD reader (Meso Scale Discovery).

The lower limit of quantitation (LLOQ) was ~34.3 ng/mL. For quantitation/evaluation, a five-parameter logistic model (5PL Marquardt, with 1/Y2 weighting) was used. A coefficient of variation percentage ≤20 and bias percentage ±20 for all STDs and QCs were defined as plate acceptance criteria.

Flow cytometry: Tumor necrosis factor-related apoptosis-inducing ligand (TRAIL) receptor occupancy was analyzed by flow cytometry (BD FACSCanto™ II, BD Biosciences, San Jose, CA, USA) using Alexa Fluor™ (AF) 647-labeled eftozanermin alfa to detect unoccupied receptors on neutrophils from all enrolled patients. Baseline staining (pre-dose) represented the binding capacity of AF 647-eftozanermin alfa to receptors. The percentage of AF 647-eftozanermin alfa–positive neutrophils pre- and post-dosing was determined.

Enzyme-linked immunosorbent assay (ELISA): Quantitative measurement of cleaved and total soluble cytokeratin 18 (M30 and M65) in plasma was performed by ELISA using the M30 Apoptosense® CK18 Kit and M65® ELISA CK18 Kit (PEVIVA®, VLVbio AB, Nacka, Sweden), respectively.

The percentage of free receptors was analyzed using a mixed-effects model, with time, dose, and time by dose interaction as fixed effects and patient as a random effect. A potential association between the maximum change of the apoptotic markers M30/M65 from baseline and eftozanermin alfa dose was evaluated. Maximum log2 fold change from baseline at various time points was used for each patient. Other pharmacodynamic and exploratory analyses are descriptive in nature.

Pre- and on-treatment tumor biopsies collection

Twenty-five patients from the dose-optimization cohort (colorectal cancer and pancreatic cancer) consented to fresh pre- and on-treatment (paired) tumor biopsies (**Online Resource 1 –** **cohort diagram**). Pre-treatment biopsies were collected anytime during the screening period – within 28 days before starting the study treatment – and on-treatment biopsies were collected 24 ± 4 h following second or third infusion. Formalin-fixed paraffin-embedded (FFPE) and flash-frozen core biopsy samples were collected. The first two cores were fixed in formalin and remaining cores were flash frozen.

Multiplex immunofluorescence

The FFPE tissue slides were stained using Opal™ Automation IHC Detection Kit (PanCK/CD4/FoxP3/DAPI) (PerkinElmer; Cambridge, MA, USA) adapted on a Leica BOND RX fully automated immunostainer. Enumeration of immune cells was evaluated using HALO® Highplex image analysis (Indica Labs, USA). Four-μm paraffin sections were deparaffinized and rehydrated, and optimized retrieval methods and staining steps were used according to the manufacturer’s instructions [1]. Whole slide imaging was performed on the PerkinElmer Vectra 3 slide scanner (PerkinElmer, Waltham, MA, USA).

RNA whole‐transcriptome sequencing was performed on RNA extracted from FFPE tissues (fresh or archival). Complementary DNA was blunt-ended, had an A base added to the 3’ ends, and had Illumina sequencing adapters ligated to the ends (Illumina, San Diego, CA, USA). Ligated fragments were then amplified for 12 cycles using primers incorporating unique index tags. Fragments were sequenced on a HiSeq 3000 using single reads extending 50 bases. Immune cell infiltration was estimated using a method similar to CIBERSORT [2] and the European Bioinformatics Institute [3].

Immunohistochemical analyses

Death receptor (DR4) immunohistochemistry (IHC) was conducted at CellCarta (formerly HistoGeneX, Antwerp, Belgium) using rabbit monoclonal antibody clone D9S1R, Cell Signaling #42533 (Danvers, MA/USA). Staining was performed on the Leica BOND RX instrument. The following three compartments were separately scored: membrane, cytoplasmic, and overall. Scoring included intensity of the staining and proportion of the cells staining at a certain intensity. Additionally, cleaved poly(ADP) ribose polymerase (c-PARP) IHC was performed (at CellCarta) using the rabbit polyclonal antibody Cell Signaling #9451 on the Ventana BenchMark XT (Ventana, Tucson, AZ, USA). PARP-positive apoptotic cells are counted in up to 15 fields at 40× by using a 5×5 counting grid, covering the entire tumor region. Only cells that are stained are classified as true cPARP-positive apoptotic cells.

Reverse phase protein array (RPPA) assessments

Samples for RPPA were prepared as described previously [4]. Either laser capture microdissection (Arcturus Bioscience, Mountain View, CA, USA) to enrich tumor epithelium or entire section was used without microdissection. Tissue lysates (diluted to 0.25 μg/mL) were printed in technical replicates onto nitrocellulose-coated glass slides. The total protein within each array spot was determined using SYPRO™ Ruby protein blot stain (Invitrogen/Molecular Probes, Eugene, OR, USA) per manufacturer’s directions and scanned using a Cy3 laser (Tecan Power Scanner, Männedorf, Switzerland). Immunostaining was performed on a Dako Autostainer per manufacturer’s instructions (CSA kit, Agilent Dako, Santa Clara, CA, USA). Additional steps were performed as previously described [5]. Phosphorylation and total levels of 52 proteins, including apoptotic family proteins and downstream signaling molecules, are shown in **Online Resource 1 –** **RPPA assessments table**.

**Pre- and on-treatment tumor biopsies: Cohort diagram**

**
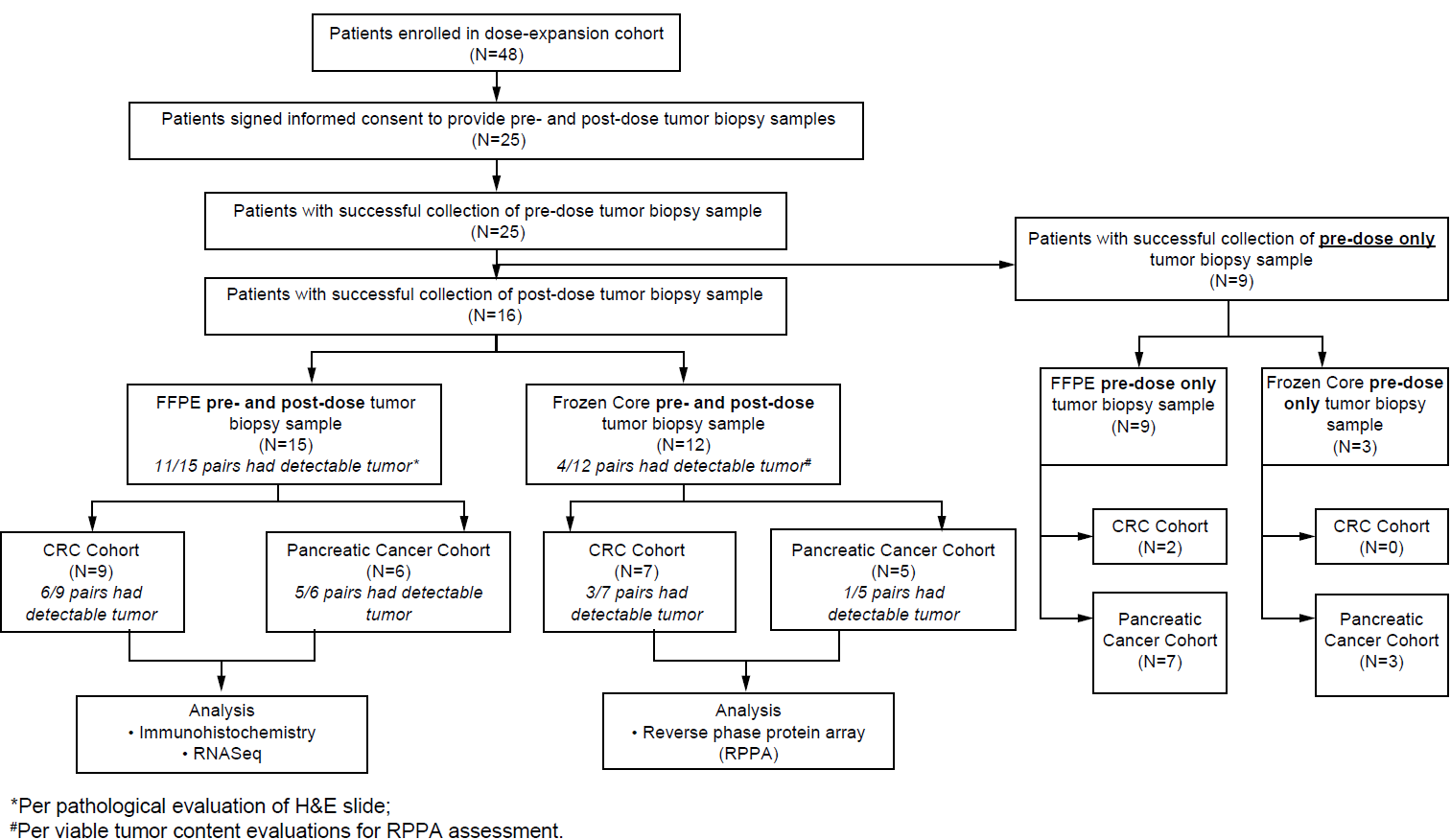
**

**RPPA assessments table**

| M30 |
| --- |
| Ki67 |
| Casp_8 |
| IkBa_S32S36 |
| IkBa |
| DR4 |
| DR5 |
| CC6_D162 |
| CC9_D330 |
| p53 |
| BIM |
| CC3_D175 |
| CC7_D198 |
| CC9_D315 |
| Cl_PARP_D214 |
| Akt_S473 |
| Akt_T308 |
| ERK_T202Y204 |
| MEK_12_S217S221 |
| FADD_S194 |
| DcR2 |
| Smac_Diablo |
| p38_MAPK_T180Y182 |
| S6_RP_S235S236 |
| NfkB_p65_p50 |
| NfkB_p65_S536 |
| NfkB_p105_S933 |
| Casp_3 |
| NfkB_p105_p50 |
| TNFa |
| Bcl2_T56 |
| Bcl2 |
| Bcl_xl |
| Bad_S136 |
| Bad_S155 |
| Bad |
| Bax |
| Mcl-1 |
| PUMA |
| Casp_7 |
| JNK_T183Y185 |
| mTOR_S2448 |
| Bak |
| NfkB_p100_p52 |
| S6_RP_S240S244 |
| Tubulin_ab |
| MEK_12 |
| Akt |
| JNK |
| Casp_9 |
| p38_MAPK |
| IL_10 |

**References**

1. Parra ER, Uraoka N, Jiang M, Cook P, Gibbons D, Forget MA, Bernatchez C, Haymaker C, Wistuba II, Rodriguez-Canales J (2017) Validation of multiplex immunofluorescence panels using multispectral microscopy for immune-profiling of formalin-fixed and paraffin-embedded human tumor tissues. Sci Rep 7:13380. https://doi.org10.1038/s41598-017-13942-8

2. Newman AM, Liu CL, Green MR, Gentles AJ, Feng W, Xu Y, Hoang CD, Diehn M, Alizadeh AA (2015) Robust enumeration of cell subsets from tissue expression profiles. Nat Methods 12:453-457. https://doi.org10.1038/nmeth.3337

3. European Bioinformatics Institute. Scientific report 2017. https://www.embl.org/files/wp-content/uploads/EMBL-EBI_Scientific_Report-2017.pdf. Accessed December 7, 2021.

4. Corcoran RB, Atreya CE, Falchook GS, Kwak EL, Ryan DP, Bendell JC, Hamid O, Messersmith WA, Daud A, Kurzrock R, Pierobon M, Sun P, Cunningham E, Little S, Orford K, Motwani M, Bai Y, Patel K, Venook AP, Kopetz S (2015) Combined BRAF and MEK inhibition with dabrafenib and trametinib in *BRAF*V600-mutant colorectal cancer. J Clin Oncol 33:4023-4031. <https://doi.org/10.1200/JCO.2015.63.2471>

5. Pierobon M, Ramos C, Wong S, Hodge KA, Aldrich J, Byron S, Anthony SP, Robert NJ, Northfelt DW, Jahanzeb M, Vocila L, Wulfkuhle J, Gambara G, Gallagher RI, Dunetz B, Hoke N, Dong T, Craig DW, Cristofanilli M, Leyland-Jones B, Liotta LA, O’Shaughnessy JA, Carpten JD, Petricoin EF (2017) Enrichment of PI3K-AKT–mTOR pathway activation in hepatic metastases from breast cancer. Clin Cancer Res 23:4919-4928. https://doi.org/10.1158/1078-0432.CCR-16-2656
