# Supplementary material for: Eftozanermin alfa (ABBV-621) monotherapy in patients with previously treated solid tumors: findings of a phase 1, first-in-human study
Source: Invest New Drugs. 2022 Apr 25;40(4):762–72. doi: 10.1007/s10637-022-01247-1 (PMC9035501; doi:10.1007/s10637-022-01247-1)
Supplement: Supplementary file 2 — Supplementary file2 (PDF 844 KB) [file 10637_2022_1247_MOESM2_ESM.pdf]

Eftozanermin alfa (ABBV-621) monotherapy in patients with previously treated solid tumors: findings of a phase 1, first-in-human study

Patricia LoRusso[1], Mark J. Ratain[2], Toshihiko Doi[3], Drew W. Rasco[4], Maja J.A. de Jonge[5], Victor Moreno[6], Benedito A. Carneiro[7], Lot A. Devriese[8], Adam Petrich[9,*], Dimple Modi[9], Susan Morgan-Lappe[9], Silpa Nuthalapati[9,*], Monica Motwani[9], Martin Dunbar[9], Jaimee Glasgow[9], Bruno C. Medeiros[9], Emiliano Calvo[10]

[1]Yale Cancer Center, New Haven, CT, USA; [2]University of Chicago, Chicago, IL, USA; [3]National Cancer Center Hospital East, Kashiwa, Japan; [4]START, San Antonio, TX, USA; [5]Erasmus MC Cancer Institute, Rotterdam, The Netherlands; [6]START Madrid-FJD, Hospital Fundación Jiménez Díaz, Madrid, Spain; [7]Cancer Center at Brown University, Lifespan Cancer Institute, Providence, RI, USA; [8]Department of Medical Oncology, University Medical Center Utrecht, Utrecht, The Netherlands; [9]AbbVie Inc., North Chicago, IL, USA; [10]START Madrid-CIOCC, Centro Integral Oncológico Clara Campal, Madrid, Spain

*Former employee of AbbVie.

Corresponding author:

Emiliano Calvo, MD, PhD

START Madrid-CIOCC

Centro Integral Oncológico Clara Campal

Hospital Madrid Norte Sanchinarro

Calle Oña, 10. 28050 Madrid, Spain

Email: emiliano.calvo@startmadrid.com

Target journal: Investigational New Drugs

Online Resource 2 – Results

Death receptor (DR)4/5 levels in archived and fresh tumor tissues

DR4 and DR5 RNA levels and DR4 protein (by immunohistochemistry [IHC]) were analyzed in archival (N=48) and fresh (before eftozanermin alfa treatment; N=32) tumor tissues from colorectal cancer (CRC) and pancreatic cancer patients from the M15-913 study (**Online Resource 2 – Fig. S3**). We were unable to identify the anti-DR5 antibody for IHC assays. The population analysis shows that the levels of DR4 and DR5 transcripts are higher in pre-treatment tumor tissues compared with archival tissue. DR4 IHC findings were also consistent with the transcripts data. Seven enrolled patients had both archival and pre-treatment tumor tissues. DR4 protein levels were higher in fresh pre-treatment tumor tissue compared with archival tissue from the same patient.

Online Resource 2 – Figure Legends

Online Resource 2 – Fig. S1 a) Reverse-phase protein array (RPPA) analysis included a panel of 50 analytes from various signaling pathways including apoptosis, MEK, AKT, etc. The subset of analytes belonging to extrinsic apoptotic pathway and downstream signaling molecules are represented on the heat map. Four of 12 patients with paired frozen cores had viable tumor cells. Two of four patients had both whole slide and laser capture microdissection analysis available. b) Analysis of paired biopsies by RPPA demonstrated modulation of multiple pathways. Increased levels of apoptotic markers M30 and cleaved poly(ADP-ribose) polymerase (c-PARP) were observed in five of six (83%) patients. Additionally, levels of pro-survival pathway molecules such as p-MEK1/2, p-AKT, and p-ERK decreased in four of six (67%), five of six (83%), and four of six (67%) patients. c) Immunohistochemical analyses in paired formalin-fixed paraffin-embedded tissues were implemented to confirm RPPA findings. c-PARP levels in pre- and on-treatment biopsies were assessed by immunohistochemistry. c-PARP protein levels increased after eftozanermin alfa administration in seven of eight (88%) patients with paired biopsies. d) Representative images of c-PARP staining in pre- (left) and on-treatment (right) biopsies. LCM, laser capture microdissection; SL, whole slide.

Online Resource 2 – Fig. S2 a) Enumeration of percentage of total tumor-infiltrating lymphocytes (TILs) in tumor cells and relative percentage of CD4-naive cell type within total TILs was performed as previously described (**Online Resource 1**) [1]. RNA sequencing data were available from 11 patients with paired formalin-fixed paraffin embedded tumor tissue. Increased TILs were observed in tumor regions of 10 of 11 (91%) patients after eftozanermin alfa administration. Within the TILs, relative CD4-naive cells percentage increased in seven of 11 (64%) patients post-eftozanermin alfa treatment. b) Increase in immune markers was confirmed using a second modality, multiplex immunohistochemistry (IHC). Multiplex IHC enumeration included assessment of CD4-positive (CD4+) cells percentage within the tumor region. Increased distribution of CD4+ cells was detected in

on-treatment samples in tumor region of six of eight (75%) patients after eftozanermin alfa treatment

Online Resource 2 – Fig. S3 Archived (N=48) and fresh biopsy tissues (N=32) collected from colorectal cancer (CRC) and pancreatic patients were tested for death receptor (DR)4, DR5 RNA by RNA sequencing (RNA-Seq), and DR4 protein by immunohistochemistry (IHC). Data from M15-913 were compared with The Cancer Genome Atlas (TCGA) (N=697). Notable caveats: TCGA samples are treatment naive. Pipeline processing of TCGA RNA-Seq data is different than M15-913. a) CRC and pancreatic cancer patients from TCGA had significantly higher expression of DR4 compared with patients enrolled in M15-913 ($p<0.0001$). b) Similarly, DR5 RNA expression was higher in TCGA tissues compared with M15-913 trial tissues. Within M15-913, DR4, DR5 RNA expression was consistently higher in fresh tissue compared with archived tissue. c) DR4 protein expression was observed to be higher in fresh biopsy vs archived tissue. FPKM, fragments per kilobase million

Online Resource 2 – Fig. S1

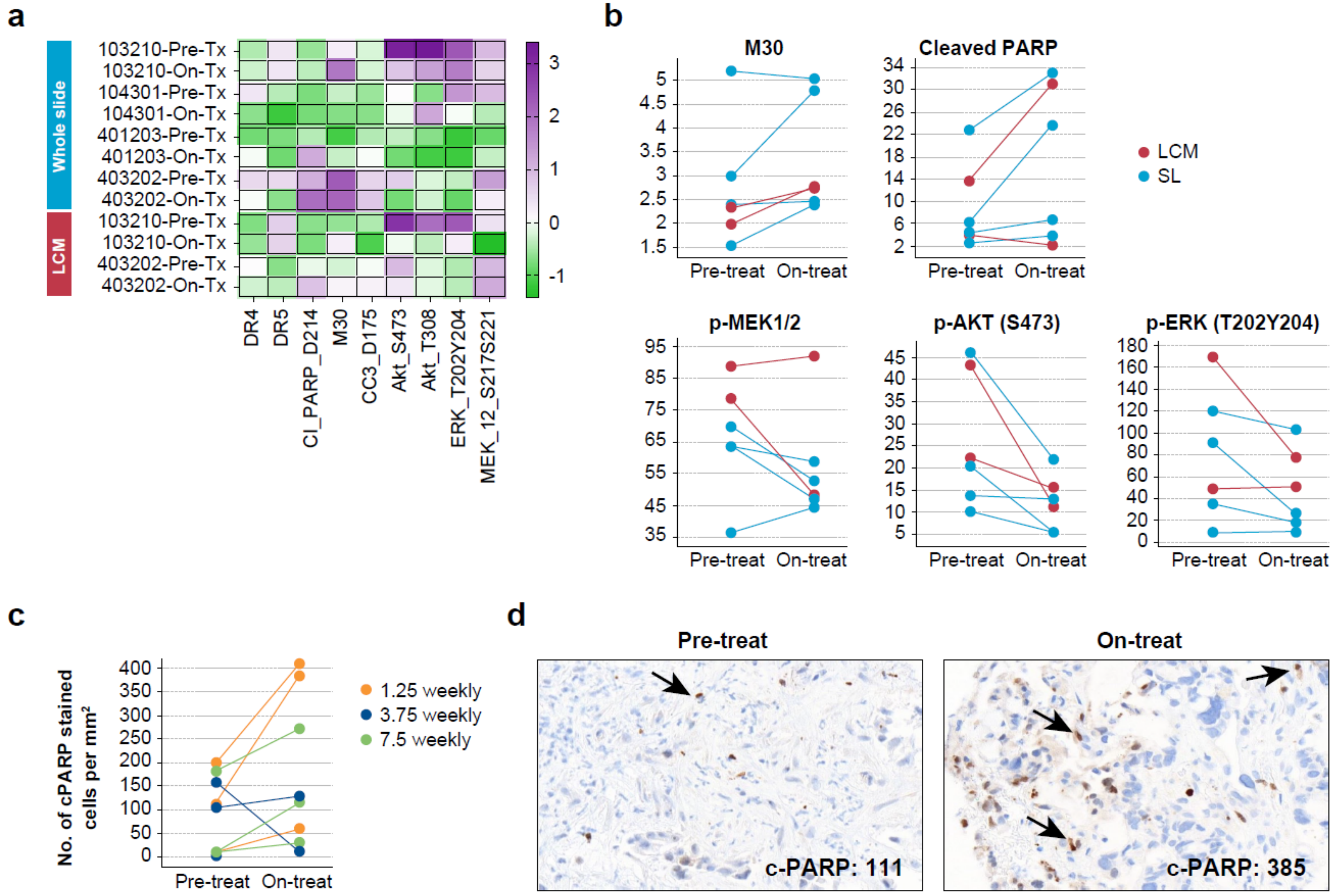

Online Resource 2 – Fig. S2

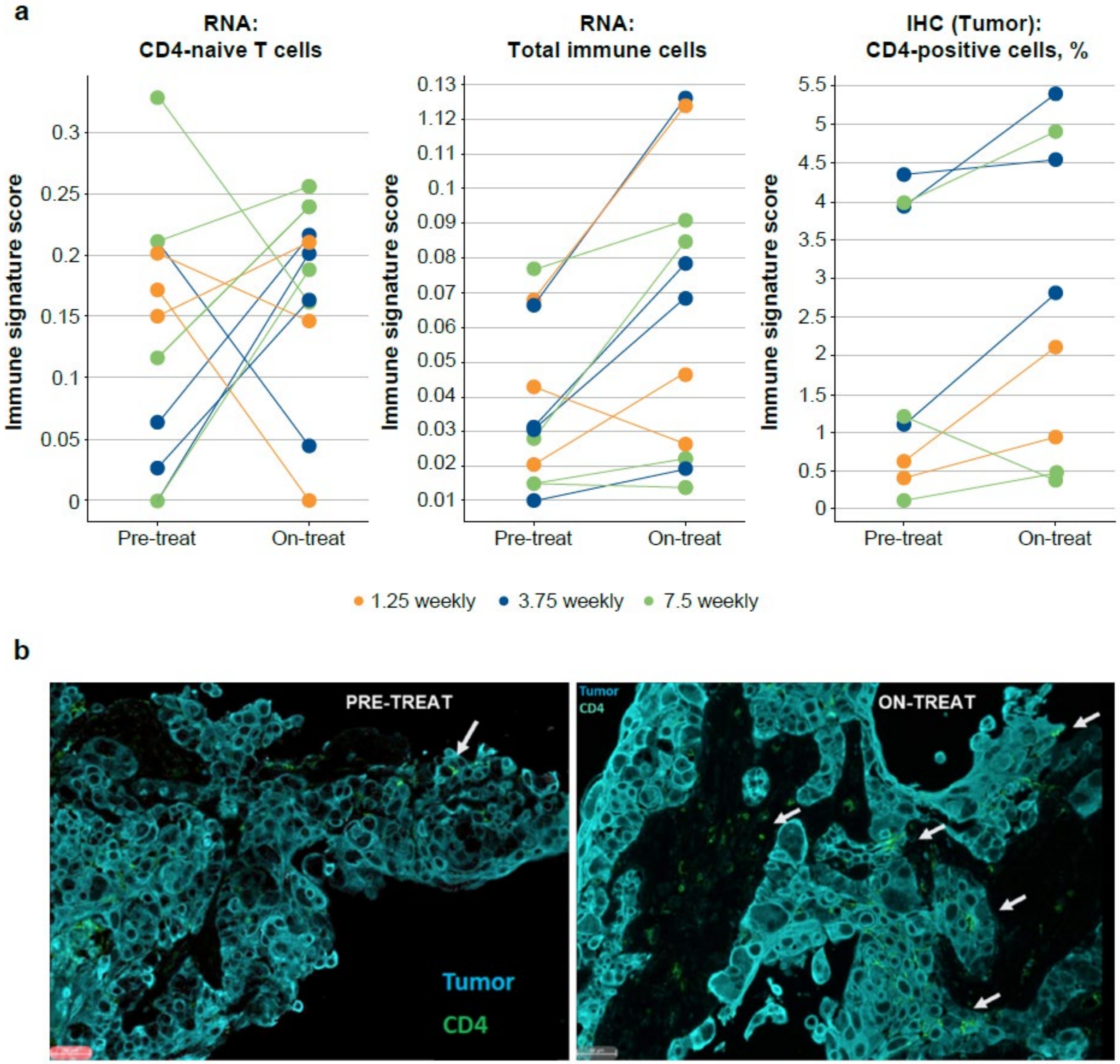

Online Resource 2 – Fig. S3

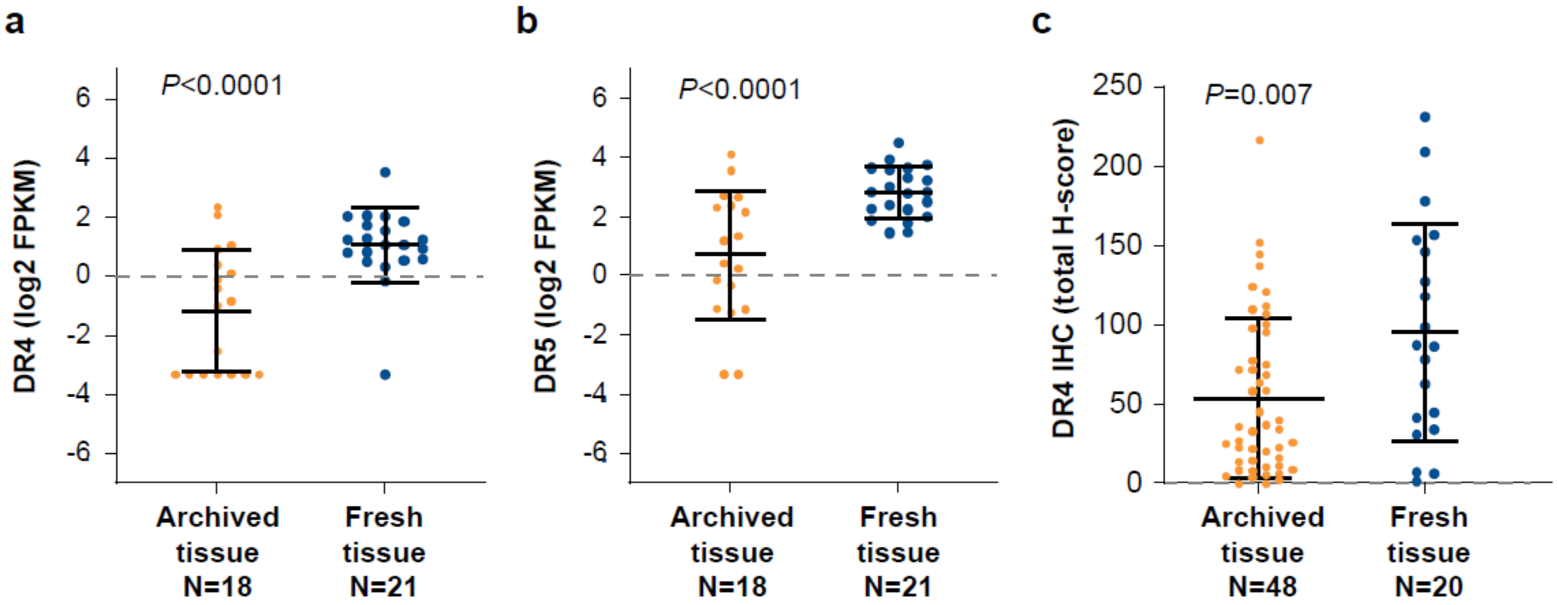

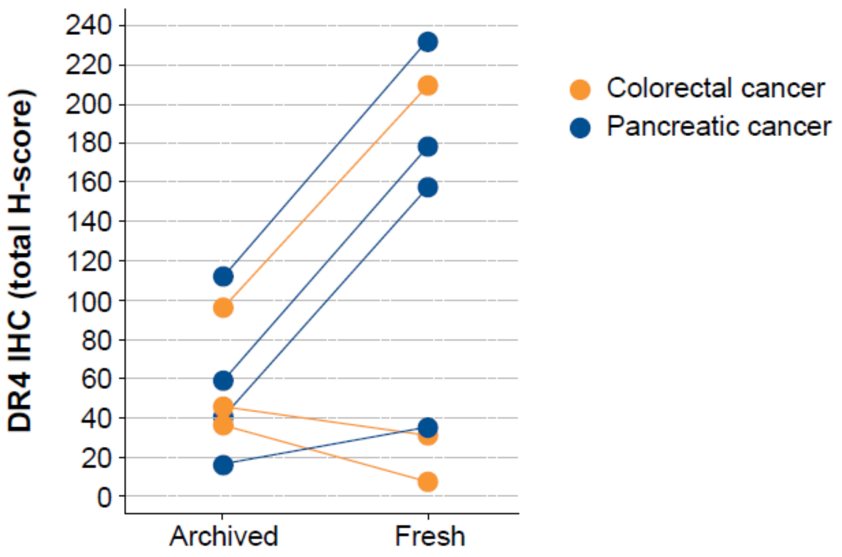

Reference

1. European Bioinformatics Institute. Scientific report 2017. https://www.embl.org/files/wp-content/uploads/EMBL-EBI_Scientific_Report-2017.pdf. Accessed December 7, 2021.
