# Supplementary material for: Eftozanermin alfa (ABBV-621) monotherapy in patients with previously treated solid tumors: findings of a phase 1, first-in-human study
Source: Invest New Drugs. 2022 Apr 25;40(4):762–72. doi: 10.1007/s10637-022-01247-1 (PMC9035501; doi:10.1007/s10637-022-01247-1)

Eftozanermin alfa (ABBV-621) monotherapy in patients with previously treated solid tumors: findings of a phase 1, first-in-human study

Patricia LoRusso[1], Mark J. Ratain[2], Toshihiko Doi[3], Drew W. Rasco[4], Maja J.A. de Jonge[5], Victor Moreno[6], Benedito A. Carneiro[7], Lot A. Devriese[8], Adam Petrich[9,*], Dimple Modi[9], Susan Morgan-Lappe[9], Silpa Nuthalapati[9,*], Monica Motwani[9], Martin Dunbar[9], Jaimee Glasgow[9], Bruno C. Medeiros[9], Emiliano Calvo[10]

[1]Yale Cancer Center, New Haven, CT, USA; [2]University of Chicago, Chicago, IL, USA; [3]National Cancer Center Hospital East, Kashiwa, Japan; [4]START, San Antonio, TX, USA; [5]Erasmus MC Cancer Institute, Rotterdam, The Netherlands; [6]START Madrid-FJD, Hospital Fundación Jiménez Díaz, Madrid, Spain; [7]Cancer Center at Brown University, Lifespan Cancer Institute, Providence, RI, USA; [8]Department of Medical Oncology, University Medical Center Utrecht, Utrecht, The Netherlands; [9]AbbVie Inc., North Chicago, IL, USA; [10]START Madrid-CIOCC, Centro Integral Oncológico Clara Campal, Madrid, Spain

*Former employee of AbbVie.

Corresponding author:

Emiliano Calvo, MD, PhD

START Madrid-CIOCC

Centro Integral Oncológico Clara Campal

Hospital Madrid Norte Sanchinarro

Calle Oña, 10. 28050 Madrid, Spain

Email: emiliano.calvo@startmadrid.com

Target journal: Investigational New Drugs

Online Resource 2

Online Resource 2 – Table S1 Dose-limiting toxicities

DLTs	Dose escalation					Dose optimization	
	2.5 mg/kg D1/D8	3.75 mg/kg D1/D8	5 mg/kg D1/D8	6.5 mg/kg D1/D8	15 mg/kg D1/D8	1.25 mg/kg QW	3.75 mg/kg QW
Patients with DLT, n (%)	1/16 (6)	3/12 (25)	1/6 (17)	1/6 (17)	1/4 (25)	3/16 (19)	1/16 (6)
DLT, n							
Increased ALT	1	1	-	1[a]	1	1[a]	1[a]
Increased AST	-	-	-	1[a]	-	1[a]	1[a]
Increased bilirubin	-	1	-	1[a]	-	-	-
Fatigue	-	1[a]	-	-	-	-	-
Nausea	-	1[a]	-	-	-	-	-
Noncardiac chest pain	-	-	-	-	-	1[b]	-
Pleuritic pain	-	-	-	-	-	1[b]	-
Respiratory failure	-	-	1	-	-	-	-
Toxic hepatitis	-	-	-	-	-	1	-

[a,b]DLTs reported in the same patient in each dose cohort.

ALT, alanine aminotransferase; AST, aspartate aminotransferase; D, day; DLT, dose-limiting toxicity; QW, once-weekly.

Online Resource 2 – Table S2 Summary of treatment-emergent and treatment-related adverse events occurring in patients with CRC and PaCA enrolled in the dose-optimization cohort

**Group**	**N**	**Treatment-emergent events, n (%)**			**Treatment-related events, n (%)**			**Treatment-related deaths**
		**TEAEs**	**Grade ≥3 TEAEs**	**SAEs**	**TRAEs**	**Grade ≥3 TRAEs**	**TRSAEs**	
Patients with ≥1 event	48	48 (100)	31 (65)	27 (56)	44 (92)	10 (21)	7 (15)	1 (2)
By dose, mg/kg								
1.25 QW	16	16 (100)	11 (69)	10 (63)	13 (81)	3 (19)	3 (19)	0
3.75 QW	16	16 (100)	11 (69)	9 (56)	15 (94)	2 (13)	1 (6)	0
7.5 QW	16	16 (100)	9 (56)	8 (50)	16 (100)	5 (31)	3 (19)	1 (6)
By indication								
CRC	24	24 (100)	13 (54)	11 (46)	22 (92)	4 (17)	1 (4)	0
PaCA	24	24 (100)	18 (75)	16 (67)	22 (92)	6 (25)	6 (25)	1 (4)

CRC, colorectal cancer; PaCA, pancreatic cancer; QW, once-weekly; TEAE, treatment-emergent adverse event; TRAE, treatment-related adverse event; TRSAE, treatment-related serious adverse event.

Online Resource 2 – Table S3 Preliminary geometric mean (mean, %CV) pharmacokinetic parameters of eftozanermin alfa[a]

Dose (mg/kg)	N	C_{max} (µg/mL)	T_{max}[b] (h)	AUC_{0-168} (µg•h/mL)	AUC_{0-inf} (µg•h/mL)	$t_{1/2}$[c] (h)	CL (mL/h/kg)
1.25 QW	11	25.8 (40.6, 161)	1.3 (1.3–3.0)	718 (733, 23)[d]	702 (722, 25)	30.6 (6.16)	1.71 (1.74, 21)[d]
3.75 D1/D8 Q3W + QW	28	59.7 (64.1, 37)	1.3 (1.3–3.0)	2080 (2160, 27)[e]	2160 (2250, 28)[e]	36.4 (5.21)[e]	1.73 (1.81, 30)[e]
7.5 QW	13	129 (132, 21)	1.3 (1.3–1.3)	3870 (3970, 24)	3990 (4100, 25)	33.4 (7.63)	1.88 (1.93, 24)
All doses	52	NA	1.3 (1.3–3.0)	NA	NA	34.1 (6.62)[f]	1.77 (1.83, 26)[g]

[a]Table reports pharmacokinetic parameters for all doses from the dose-optimization cohorts; 3.75 mg/kg summarizes pharmacokinetics from both dose-escalation and dose-optimization cohorts.
[b]Median (minimum–maximum).
[c]Harmonic mean (pseudo-standard deviation).
[d]N=10.
[e]N=25.
[f]N=49.
[g]N=48.
AUC_{0-168}, area under the serum concentration-time curve from time zero to 168 h after dosing; AUC_{0-inf}, area under the serum concentration-time curve from time zero to infinite time; CL, clearance; C_{max}, maximum observed serum concentration; CV, coefficient of variation; D, day; N, total sample size; NA, not applicable; QW, once-weekly; Q3W, every 3 weeks; $t_{1/2}$, terminal phase elimination half-life; T_{max}, time to maximum observed serum concentration, peak time.

Online Resource 2 – Fig. S1 Disposition of patients enrolled in the dose-optimization cohort according to dosing or disease group, and overall disposition. CRC, colorectal cancer; PaCA, pancreatic cancer

**Dose or disease cohort**<br>Number of patients	**1.25 mg/kg**<br>n=16	**3.75 mg/kg**<br>n=16	**7.5 mg/kg**<br>n=16	**CRC total**<br>n=24	**PaCA total**<br>n=24	**All patients**<br>N = 48
**Median (range) time on treatment, days**	**37 (1–232)**	**43 (1–290)**	**36 (15–254)**	**40 (1–290)**	**36 (1–288)**	**36 (1–290)**
Median no. of cycles (range)*	2 (1–12)	3 (1–14)	2 (1–13)	3 (1–14)	2 (1–14)	2 (1–14)
**Eftozanermin alfa discontinuation, n (%)**						
Adverse event	1 (6)	2 (13)	2 (13)	3 (13)	2 (8)	5 (10)
Progressive disease	13 (81)	12 (75)	13 (81)	19 (79)	19 (79)	38 (79)
Consent withdrawal	0	0	0	0	0	0
Physician decision	2 (13)	1 (6)	1 (6)	2 (8)	2 (8)	4 (8)
Death	0	0	0	0	0	0
Other	0	1 (6)	0	0	1 (4)	1 (2)
	Disposition according to dosing cohort			Disposition according to disease cohort		Disposition overall

*All patients received ≥1 cycle. CRC, colorectal cancer; PaCA, pancreatic cancer.

Online Resource 2 – Fig. S2 Spider plot of percentage change in size of tumor lesions over time in patients with pancreatic cancer with one or more post-baseline tumor assessment (dose-optimization cohort). Baseline tumor assessments were performed at D1 (baseline), within 28 days of C1D1, within 7 days before dosing on C3D1 (post-baseline), and Q9W thereafter. C, cycle; D, day; Q9W, every 9 weeks

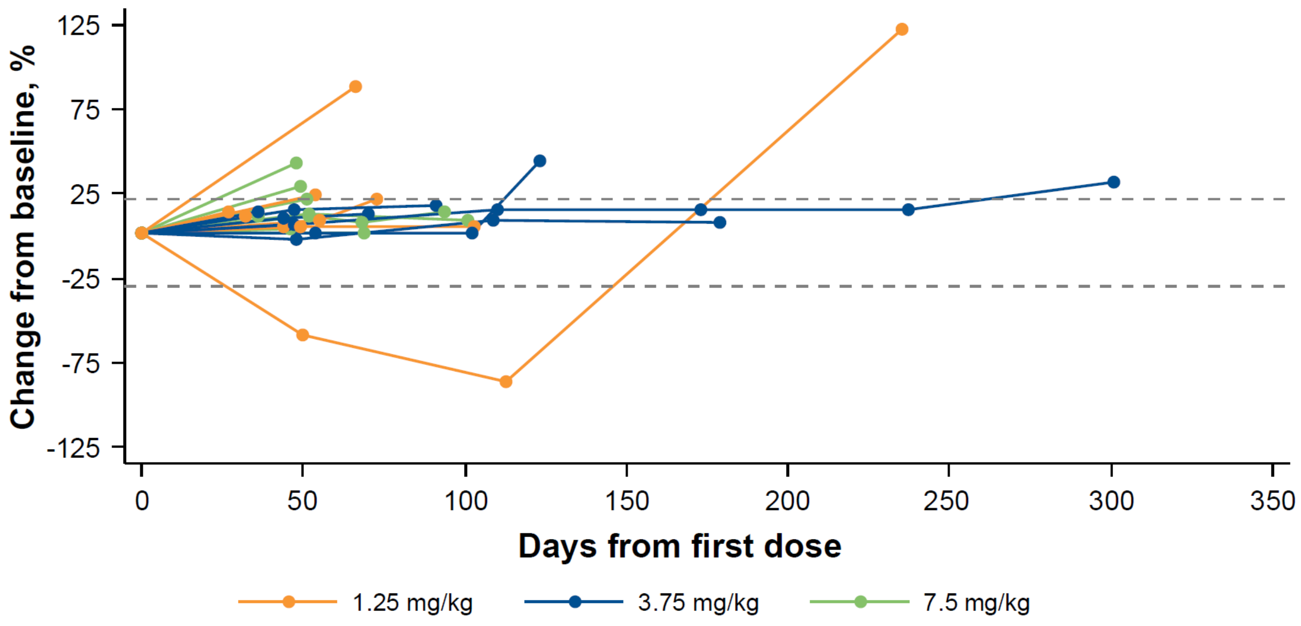

Online Resource 2 – Fig. S3 Mean fold change (± SD) in serum M30 (a) and M65 (b) levels in patients with pancreatic cancer treated with eftozanermin alfa in the dose-optimization cohort. C, cycle; D, day; HR, hour; PRE, pre-dose; QW, once-weekly; SD, standard deviation

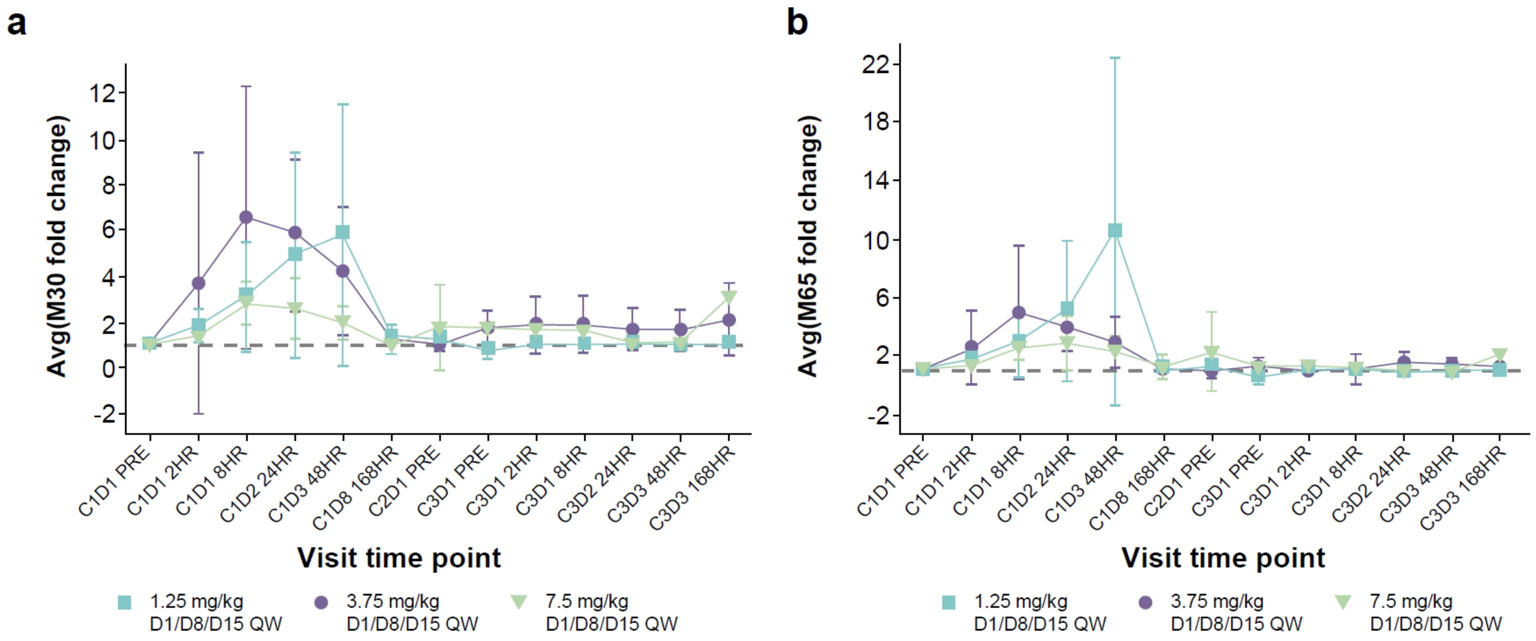

Supplement: Supplementary file 3 — Supplementary file3 (PDF 464 KB) [file 10637_2022_1247_MOESM3_ESM.pdf]
